# Association between metallic implants and stroke in US adults from NHANES 2015–2023 a cross-sectional study

**DOI:** 10.3389/fnagi.2024.1505645

**Published:** 2024-12-20

**Authors:** Kai Wu, Liang Pang, Pingping Su, Cunxian Lv

**Affiliations:** Wenzhou TCM Hospital of Zhejiang Chinese Medical University, Wenzhou, China

**Keywords:** metal implants, stroke, cross-sectional study, NHANES, risk factors

## Abstract

**Objective:**

Metal implants play a vital role in orthopedic treatment and are widely used in fracture repair, joint replacement and spinal surgery. Although these implants often contain key elements such as chromium (Cr), their potential health effects, particularly their association with stroke risk, have not been fully elucidated. This study aimed to evaluate the association between metallic implants and stroke.

**Methods:**

Using data from the 2015 to 2023 National Health and Nutrition Examination Survey (NHANES), we conducted a cross-sectional analysis of 12,337 US adults, in which 3,699 participants reported having metal implants and 8,638 without. Implant-like.

**Results:**

Through logistic regression analysis, we revealed a significant positive association between metallic implants and stroke risk (adjusted OR = 1.458, 95%CI (1.130, 1.881), *p* = 0.004). Further stratified analysis found that this positive association was more significant among older and less physically active participants.

**Conclusion:**

Findings suggest that metallic implants may be associated with an increased risk of stroke, but given the inherent limitations of cross-sectional studies, this study cannot establish causality.

## 1 Introduction

Stroke, clinically referred to as cerebrovascular accident, is a syndrome characterized by brain dysfunction resulting from pathological events in the brain’s blood vessels such as arterial embolism, small vessel disease, or cerebral infarction. Clinically, it primarily manifests as two types: hemorrhagic and ischemic, with the latter being more predominant in terms of incidence (Sacco et al., 2013; Rost et al., 2022; Kumar et al., 2010). Global burden of disease studies have shown that stroke is one of the leading causes of death and disability worldwide, posing a significant economic burden (Wafa et al., 2020). With the aging population, the prevalence of stroke is expected to rise in the coming decades, exacerbating economic and social pressures. Numerous risk factors contribute to stroke, including but not limited to age, sex, genetic predisposition, and lifestyle factors, among which chronic diseases and unhealthy habits are recognized as critical risk factors (GBD 2019 Stroke Collaborators, 2021; Roerecke, 2021).

Joint replacement surgery, particularly hip and knee replacements, has become an established effective method for treating advanced joint pain and functional impairment (Maradit et al., 2015). Given the global trend of population aging, it is projected that by 2040, the number of joint replacement surgeries performed annually in the United States will approach 5 million (Singh et al., 2019). While joint replacement surgeries provide significant symptom relief and functional recovery, postoperative complications such as neurovascular injuries, deep vein thrombosis (DVT), and prosthetic infections should not be overlooked (Santana et al., 2020; Vrgoc et al., 2014). Furthermore, clinical studies have indicated that patients undergoing total hip replacement face a heightened risk of stroke (Rasouli et al., 2016; Richardson et al., 2023; Pang et al., 2024). Joint prostheses are often made from metal alloys, including cobalt-chromium alloys, stainless steel, titanium alloys, as well as biocompatible materials like ceramics and polymers (Manthe et al., 2022). These metal implants endure mechanical stress and the body’s fluid environment (such as blood, tissue fluid, and lymphatic fluid) over the long term, potentially leading to localized and systemic release of metal ions (He et al., 2023). Components in the body fluid environment, such as electrolytes (such as sodium ions and chloride ions), proteins, and other organic molecules, can affect the corrosion behavior of the metal surface through chemical and electrochemical reactions, leading to the release of metal ions. Notably, cobalt has been shown to have potential cardiotoxic effects, with significant increases in cobalt concentrations in myocardial cells following total hip replacement (Wyles et al., 2017; Cheung et al., 2016; Berber et al., 2017).

Based on this background, the present cross-sectional study aims to utilize data from the National Health and Nutrition Examination Survey (NHANES) to explore the relationship between metal implants and stroke risk among U.S. adults. We hypothesize that individuals with metal implants have a higher risk of stroke compared to those without such implants. The findings of this study are expected to provide new scientific evidence regarding the association between metal implants and stroke risk, offering theoretical support for clinical decision-making, patient counseling, and public health policy formulation.

## 2 Materials and methods

### 2.1 Study population

**Figure 1:**
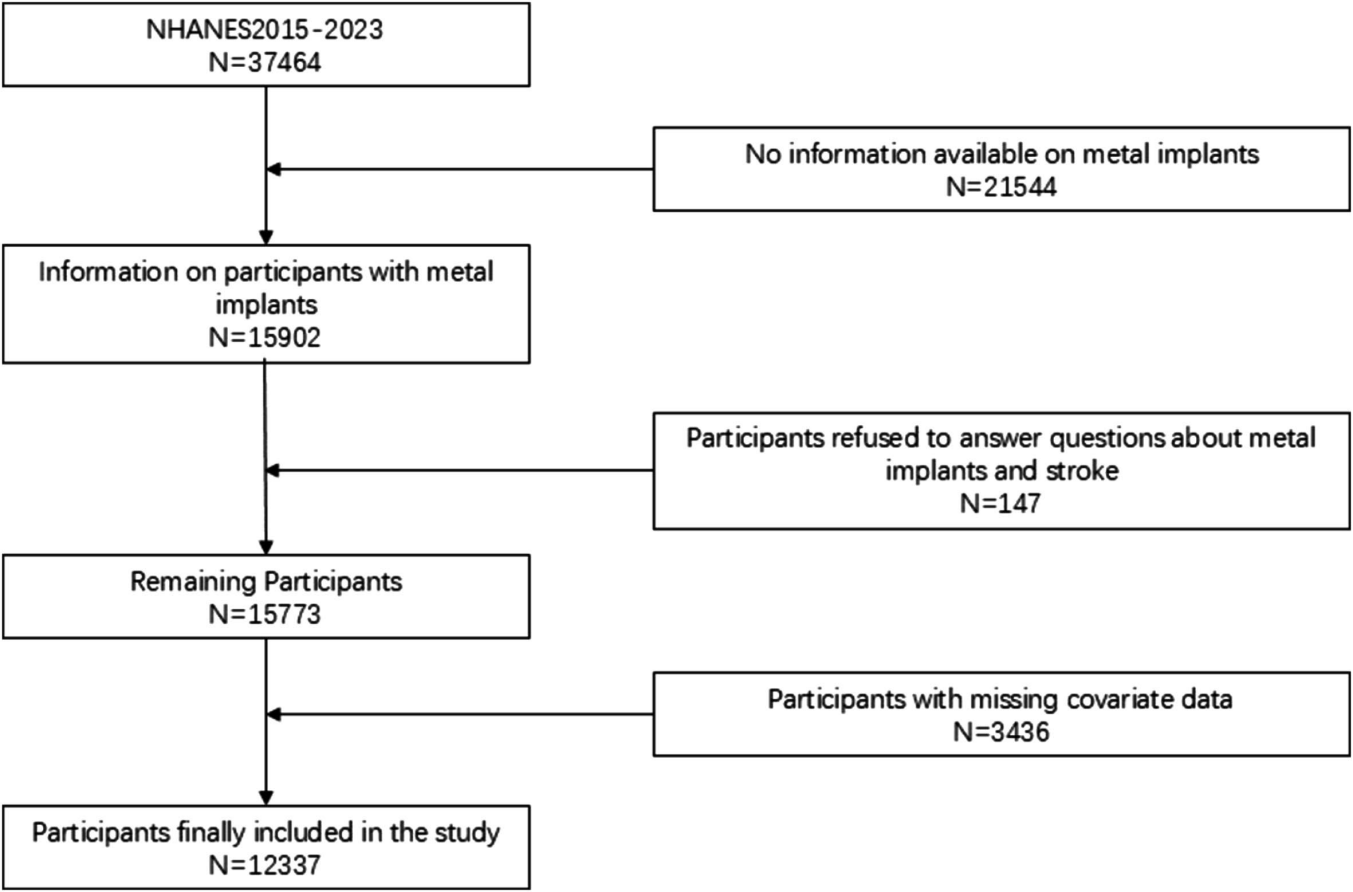
Flowchart of participant selection.

The National Health and Nutrition Examination Survey (NHANES) is a nationally representative survey conducted across the United States, employing random sampling to select participants, ensuring that the data is representative of the entire U.S. population. Implemented biennially, each cycle of the survey encompasses face-to-face interviews, physical examinations, laboratory tests, and other health measurements for thousands of residents. The data from NHANES are extensively utilized for formulating public health policies, guiding research, and evaluating national health objectives. It should be noted that, while the NHANES data provide nationally representative information, the study was unable to analyze potential regional differences due to the inability to obtain specific geographic area data. This may limit the extrapolation of the results to certain specific areas. However, this limitation does not detract from the national applicability of the results, as the sampling method of NHANES ensures broad representativeness of the data. Future studies can combine other databases with geographic area details for further exploration. A total of 37,464 participants were obtained in the selected NHANES cycle. We further excluded subjects with missing questionnaire data and relevant laboratory tests. Ultimately, a total of 12,337 participants were included in this study. Figure 1 illustrates the flowchart for participant selection.

### 2.2 Measurement and definition

The presence of metal implants in the body was determined based on an affirmative response to the following question: “Do you have any artificial joints, pins, plates, metal sutures, or other types of metal objects in your body?” Additionally, the mentioned metal implants do not include piercings, dental crowns, braces or fixtures, shrapnel, or bullets, and should not be visible externally or in the mouth. Stroke diagnosis was further assessed using a health questionnaire. During the interview, participants were asked, “Have you ever been diagnosed with a stroke by a doctor or other healthcare professional?” Participants who answered “yes” to this question were considered stroke survivors. This definition has been used in numerous epidemiological studies and has been effective in capturing health events among participants. However, due to the lack of radiological confirmation or medical records, future studies should employ a variety of methods to verify the accuracy of stroke diagnoses.

### 2.3 Covariates

In line with previous studies, we collected variables that may be associated with the relationship between metallic implants and stroke, including smoking, alcohol consumption, obesity, and diabetes. We obtained sociodemographic data (such as gender, age, race/ethnicity, poverty status [PIR], education level, and body mass index [BMI]), lifestyle factors (such as smoking and drinking status), and medical history (such as diabetes) as covariates (He et al., 2023). Education levels were categorized into three groups used by NHANES: high school or below, high school equivalent, and college or above. Participants were classified as Mexican American, other Hispanic, Hispanic white, non-Hispanic black, or other races. The PIR was divided into three categories: low (<1.5), medium (1.5–3.5), and high (>3.5). Marital status was defined as one of three groups: married and living with a partner, other (including widowed, divorced, or separated), and never married. For lifestyle factors, individuals who reported having smoked more than 100 cigarettes in their lifetime were classified as smokers. Alcohol consumption was assessed through the questions: “During the past year, have you consumed at least 12 drinks of any kind of alcoholic beverage? By “one drink,” I mean a 12-ounce beer, a 5-ounce glass of wine, or a 1.5-ounce shot of spirits?” (Liu et al., 2022). Diabetes was defined as self-reported diabetes, use of antidiabetic medications, or meeting the criteria set by the American Diabetes Association (ADA). Participants were classified as having hypertension if they met any of the following criteria: (Sacco et al., 2013) average systolic blood pressure (SBP) ≥ 140 mmHg (Rost et al., 2022) average diastolic blood pressure (DBP) ≥ 90 mmHg (Kumar et al., 2010) self-reported diagnosis of hypertension; or (Wafa et al., 2020) currently using antihypertensive medications. Coronary heart disease was determined based on a questionnaire asking participants whether a doctor had ever informed them of this condition.

### 2.4 Statistical analysis

Statistical analyses were conducted using Stata 16 and R software version 4.2.1. Descriptive statistics were employed to present the data, with normally distributed or skewed data reported as mean ± standard deviation, and categorical variables expressed as counts and percentages. Complex sample weights were utilized to estimate population characteristics, BMI, and the overall prevalence of diabetes and hypertension.

Subsequently, statistically significant covariates were incorporated into a multiple linear regression model for analysis. In this study, metal implants served as the predictor variable, while stroke was the outcome variable for the association study. After adjusting for covariates, the relationship between metal implants and stroke was further explored, and effect sizes (*β*) along with their 95% confidence intervals (CI) were calculated. To enhance the accuracy of the results, subgroup analyses stratified by age, gender, and physical activity were performed. A *p*-value of <0.05 was considered statistically significant for assessing the performance of the metrics.

## 3 Results

### 3.1 Population-based research analysis

A total of 12,337 eligible participants were included in this study, comprising a metal implant group (*n* = 3,699) and a non-metal implant group (*n* = 8,638). The average age of participants was 58.570 years (standard error ± 11.565), with 47.624% being male. Among them, 753 individuals had a history of stroke. Table 1 presents the demographic and baseline characteristics of the metal implant and non-metal implant groups. Notably, the metal implant group was significantly older than the non-metal implant group (*p* < 0.001). The proportion of non-Hispanic whites in the metal implant group was higher than in the non-metal implant group (75.621 vs. 63.940%).

**Table 1:** Baseline characteristics of participants in the NHANES 2015–2020 cycle.

Characteristics	Total	Without metal implants	With metal implants	*p*
Age (years), Mean (SD)	58.570 + 11.565	56.662 + 11.114	62.848 + 11.412	<0.001
Total cholesterol (mg/dL), Mean (SD)	194.529 + 42.677	197.350 + 42.001	188.204 + 43.495	<0.001
HDL (mg/dL), Mean (SD)	55.257 + 17.289	55.044+ 16.686	55.732 + 18.559	0.041
Gender, (%)				0.304
Man	47.624	47.933	46.933	
Female	52.376	52.067	53.067	
Race/ethnicity, *n* (%)				<0.001
Mexican American	6.677	7.602	4.603	
Other Hispanic	6.706	7.414	5.119	
Non-Hispanic White	67.543	63.940	75.621	
Non-Hispanic Black	9.851	11,168	6.899	
Other Race	9.222	9.876	7.758	
Education level, (%)				0.065
High school graduate or below	36.752	37.012	36.169	
Some college or AA degree	30.492	29.850	31.931	
College graduate or above	32.756	33.138	31.900	
Family income, (%)				0.0004
≤ 1.3	16.788	17.669	14.812	
1.3 < PIR ≤ 3.5	35.177	34.870	35.866	
PIR > 3.5	48.035	47.461	49.322	
Marriage status, (%)				0.566
Married or living with a partner	69.186	69.271	68.994	
Widowhood, divorce or separation	28.480	28,311	28.859	
Never married	2.334	2.418	2.146	
Drinking status, (%)				0.928
No	23.547	23.524	23.599	
Yes	76.453	76.476	76.401	
BMI, (%)				0.004
Normal weight	23.729	24.530	21.933	
Overweight	33.485	33.406	33.664	
Obesity	42.786	42.064	44.404	
Diabetes, (%)				<0.001
No	83,271	85.110	79.146	
Yes	16.729	14.890	20.854	
Hypertension, (%)				<0.001
No	49.341	52.766	41.664	
Yes	50.659	47.234	58.336	
Physical activity, (%)				<0.001
Regular activities	16.239	17.639	13.100	
Occasional activities	33.429	32.997	34.399	
Little activity	50.332	49.364	52.501	
Coronary heart disease, (%)				<0.001
No	94.042	97.359	86.603	
Yes	5.958	2.641	13.397	
Smoking status, (%)				<0.001
No	54.702	57.409	48.634	
Yes	45.298	42.591	51.366	
Stroke, (%)				<0.001
No	95.232	96.403	92.605	
Yes	4.768	3.597	7.395	

Furthermore, the metal implant group exhibited higher rates of poverty, lower education levels, smoking, alcohol consumption, BMI, hypertension, coronary heart disease, physical inactivity, and diabetes (all *p* < 0.05). Importantly, the prevalence of stroke was higher in the metal implant group compared to the non-metal implant group (7.395 vs. 3.597%, *p* < 0.001).

Table 2 presents the logistic regression analysis with metal implants as the independent variable and stroke as the outcome. Overall, metal implants were significantly associated with stroke [OR = 2.140, 95% CI (1.720, 2.663), *p* < 0.001]. This positive correlation persisted even after adjusting for other variables, remaining significant in Model 2 [OR = 1.835, 95% CI (1.463, 2.301)], Model 3 (OR = 1.650, 95% CI (1.305, 2.088), *p* < 0.001), and Model 4 (OR = 1.458, 95% CI (1.130, 1.881), *p* = 0.004). In all models, the presence of metal implants was positively correlated with the incidence of stroke, and this association remained statistically significant.

**Table 2:** Relationship between metal implants and stroke.

		OR (95%CI)	*p*
Model 1	No stroke	ref	
	Stroke	2.140 (1.720, 2.663)	<0.001
	No stroke	ref	
Model 2	Stroke	1.835 (1.463, 2.301)	<0.001
	No stroke	ref	
Model 3	Stroke	1.650 (1.305, 2.088)	<0.001
	No stroke	ref	
Model 4	Stroke	1.458 (1.130, 1.881)	0.004

Table 3 illustrates the results of the stratified analysis, which demonstrates that the correlation between metal implants and stroke increases with age and decreases in physical activity. The relationships in the final model are as follows: for the >60 age group [OR = 1.513, 95% CI (1.129, 2.028), *p* = 0.006], for the group with low physical activity [OR = 1.382, 95% CI (1.043, 1.829), *p* = 0.024], and for women [OR = 1.382, 95% CI (1.043, 1.829), *p* = 0.024].

**Table 3:** Subgroup analysis between metal implants and stroke subgroup analysis of the relationship between metallic implants and stroke.

Subgroup	Variable	OR (95%CI)	p
Age	≤60	1.282 (0.784, 2.095)	0.322
	>60	1.513 (1.129, 2.028)	0.006
Gender	Man	1.293 (0.904, 1.851)	0.160
	Female	1.649 (1.167, 2.332)	0.005
Physical activity	Regular activities	1.970 (0.627, 6.186)	0.245
	Occasional activities	1.553 (0.864, 2.792)	0.141
	Little activity	1.382 (1.043, 1.829)	0.024

Each stratification factor was adjusted for all other variables except the stratification component itself.

## 4 Discussion

This study utilized nationally representative data from the NHANES (2015–2023) to elucidate the strong association between metal implants and stroke. Importantly, this association remained statistically significant even after adjusting for several confounding factors, including age, gender, race, physical activity, socioeconomic status, obesity, smoking status, alcohol consumption, hypertension, and diabetes. In our research, approximately 7.395% of patients in the metal implant group had a history of stroke, which is 3.798% higher than that in the non-metal implant group. These findings prompt us to consider the possibility that metal implants may be a potential risk factor for stroke. In subgroup analysis, it has been observed that women who undergo metal implantation are at an increased risk of stroke compared to their male counterparts. Research indicates that women tend to have higher concentrations of metal ions in their bodies (Moroni et al., 2011). Furthermore, individuals over the age of 60 are at a higher risk of developing stroke, and those with less physical activity also face an elevated risk. Studies have shown that the proportion of non-Hispanic whites in the group with metal implants is significantly higher than that of other ethnicities, which may reflect differences in access to medical resources, surgical preferences, and cultural factors among different ethnic groups. For instance, research has indicated that non-Hispanic whites are more inclined to undergo joint replacement surgery or other treatment measures involving metal implants (Kim and Jacobson, 2024; Jiang et al., 2024). This racial distribution difference may suggest that racial factors play a significant role in the association between metal implants and stroke risk. Therefore, this distribution characteristic emphasizes the importance of balancing racial representation in future study designs.

Previous studies have primarily focused on the release of metal ions due to wear or corrosion of metal implants. For instance, some researchers have identified mechanical-assisted crevice corrosion (MACC) following total hip arthroplasty, during which metal ions may enter the bloodstream (Garbuz et al., 2010; Jacobs et al., 2014; Hussey and McGrory, 2017). The accumulation of these ions in the body can be disseminated through circulation, potentially affecting overall health, including vascular health (Brennan et al., 2024; Lassalle et al., 2018; Mastel et al., 2022). Although there is currently no direct evidence linking metal ions to stroke causation, studies have indicated that the normal functioning of brain tissue relies on essential ion transporters. The accumulation of ions may disrupt the operation of these transporters, potentially leading to a vicious cycle that increases stroke risk (Song et al., 2024; Wang et al., 2024; Sangani et al., 2024). Additionally, research has shown that cobalt ions can lead to apoptosis and necrosis of endothelial cells once they diffuse into the bloodstream, thereby inhibiting the activity of other cells and increasing stroke risk (Zhu et al., 2021). The potential effects of metal ions on cardiac function, including disruption of heart rhythms and impairment of valve function, may also indirectly elevate the risk of stroke. One study observed that cobalt ions exhibited cytotoxic effects on synovial cells (fibroblasts), causing inflammation in hips with metal implants (Eltit et al., 2017; Eltit et al., 2021). Furthermore, cobalt ions can affect mitochondrial function, leading to autophagy of mitochondria and triggering hypoxic responses (Eltit et al., 2019; Chamaon et al., 2019). Hypoxic responses in cells include the secretion of cytokines that can provoke inflammation by activating vasculature and enhancing leukocyte migration (Mittal et al., 2014). Metal ions may also have widespread effects on biological systems by inducing oxidative stress responses. For instance, studies have shown that titanium dioxide nanoparticles can trigger oxidative stress, leading to repeated cellular damage and abnormal proliferation, while also causing indirect DNA damage (Zhong et al., 2024; Braakhuis et al., 2021). These processes may lead to abnormal cell growth behavior and further promote osteolysis around the prosthesis (Nuevo-Ordonez et al., 2011). In addition, some studies have found that increased titanium concentrations in plasma are significantly associated with an increased risk of stroke and heart disease (Xiao et al., 2019; Yuan et al., 2017).

Furthermore, metal allergic reactions are also a concern following joint replacement surgery. Some patients may develop allergic reactions to certain components in metal implants, such as cobalt and chromium, potentially leading to local tissue reactions or even systemic responses. In a study by Bloemke and Clarke (2015), a self-reported survey on skin allergies, metal allergies, or sensitivities was conducted among patients undergoing primary total knee arthroplasty (TKA), revealing that individuals with these allergic symptoms comprised as much as 14% of the sample. In contrast, Nam et al. found a self-reported incidence of metal allergies at 4.1% through analysis of a large cohort (Nam et al., 2016). Additionally, the immune response triggered by metal allergies may indirectly affect cardiac function, increasing the risk of arrhythmias and changes in blood composition, all of which could promote the occurrence of strokes (Lv et al., 2024). Therefore, although direct evidence is lacking, the potential link between metal allergies and stroke should not be overlooked, necessitating further research to explore these underlying mechanisms and provide patients with more comprehensive prevention and treatment strategies.

Research indicates that levels of physical activity may play a significant modulating role between metal implants and the risk of stroke. Particularly in groups with low physical activity, the positive correlation between metal implants and stroke risk is more pronounced, which may be related to the hemodynamic changes and decreased metabolic levels triggered by insufficient physical activity (Belfiore et al., 2018; Ju et al., 2024; Madsen et al., 2022). Furthermore, studies have shown that replacing 1 h of sedentary time (ST) with an equivalent amount of physical activity (PA) per week can significantly reduce the risk of all-cause mortality, cardiovascular disease, and cancer deaths (de la Camara et al., 2024; Schmid et al., 2016; Rees-Punia et al., 2019). Metal ions in the body can promote vascular pathology by activating inflammatory factors. However, moderate physical activity can not only neutralize the potential side effects of metal ions but also improve overall prognosis by raising the patient’s health threshold (Tian et al., 2022; You et al., 2023; Kong et al., 2023). In contrast, although high-intensity physical activity may increase joint load and exacerbate implant wear, thereby releasing more metal ions, appropriate physical activity helps to balance this risk. It is worth mentioning that a study found that moderate physical activity can also reduce the levels of heavy metal ions such as cadmium and lead in the blood, thereby alleviating their toxic effects on the vascular system and further reducing the potential risk of stroke (Deng et al., 2024). This evidence suggests that physical activity not only has a significant impact on the biological effects of implant wear and metal ion release but may also indirectly reduce the risk of stroke by modulating inflammatory responses and improving vascular function.

In summary, although the direct relationship between metal implants and stroke following joint replacement surgery has yet to be definitively established, the release of metal ions, metal allergic reactions, and the necessity for long-term health monitoring in patients represent key areas for future research. Future studies should explore how these factors collectively impact patients’ long-term health outcomes and their potential influence on stroke risk.

Additionally, the NHANES database lacks detailed records regarding metal implants, including the type, number, duration within the body, and key information such as the corrosion rates and ion release profiles of different metal materials. This data absence limits a comprehensive assessment of the potential impact of different metal types on stroke risk. Moreover, significant differences may exist in functionality, biomechanical load, and *in vivo* degradation processes of different metal implants, which could influence study outcomes, increase heterogeneity between groups, and reduce the clinical specificity of study conclusions. Lastly, a limitation of this study is the significant difference in racial distribution between the metal implant group and the non-implant group, which may affect the universality of the results. Although we adjusted for racial factors in multivariate regression analysis, this imbalanced distribution may still mask specific associations within certain subgroups. Future research will aim to enhance clinical relevance and specificity by collecting more detailed information regarding metal implants and addressing potential confounding factors. To overcome these limitations, prospective study designs should be employed in future research. Moreover, we must acknowledge the possibility of unmeasured or unknown confounding factors, which could lead to residual confounding effects in the study.

## Data Availability

The original contributions presented in the study are included in the article/supplementary material, further inquiries can be directed to the corresponding author.

## References

[ref1] Belfiore P. Miele A. Galle F. Liguori G. (2018). Adapted physical activity and stroke: a systematic review. J. Sport Med. Phys. Fit 58. doi: 10.23736/S0022-4707.17.07749-0, PMID: 29072029

[ref2] Berber R. Abdel-Gadir A. Rosmini S. Captur G. Nordin S. Culotta V.. (2017). Assessing for cardiotoxicity from metal-on-metal hip implants with advanced multimodality imaging techniques. J. Bone Joint Surg. Am. 99, 1827–1835. doi: 10.2106/JBJS.16.00743, PMID: 29088037 PMC6948834

[ref3] Bloemke A. D. Clarke H. D. (2015). Prevalence of self-reported metal allergy in patients undergoing primary total knee arthroplasty. J. Knee Surg. 28, 243–246. doi: 10.1055/s-0034-1381959, PMID: 24949984

[ref4] Braakhuis H. M. Gosens I. Heringa M. B. Oomen A. G. Vandebriel R. J. Groenewold M.. (2021). Mechanism of action of TiO(2): recommendations to reduce uncertainties related to carcinogenic potential. Annu. Rev. Pharmacol. 61, 203–223. doi: 10.1146/annurev-pharmtox-101419-100049, PMID: 32284010

[ref5] Brennan P. C. Peterson S. M. O'Byrne T. J. Laporta M. L. Wyles C. C. Jannetto P. J.. (2024). Blood metal concentrations and cardiac structure and function in total joint arthroplasty patients. World J. Orthop. 15, 773–782. doi: 10.5312/wjo.v15.i8.773, PMID: 39165877 PMC11331322

[ref6] Chamaon K. Schonfeld P. Awiszus F. Bertrand J. Lohmann C. H. (2019). Ionic cobalt but not metal particles induces ROS generation in immune cells in vitro. J. Biomed. Mater. Res. B 107:1246:53. doi: 10.1002/jbm.b.34217 30261124

[ref7] Cheung A. C. Banerjee S. Cherian J. J. Wong F. Butany J. Gilbert C.. (2016). Systemic cobalt toxicity from total hip arthroplasties: review of a rare condition part 1 - history, mechanism, measurements, and pathophysiology. Bone Joint J. 98-B, 6–13. doi: 10.1302/0301-620X.98B1.36374, PMID: 26733509

[ref8] de la Camara M. A. Ortiz C. Granero-Melcon B. Martinez-Portillo A. Neira-Leon M. Galan I. (2024). Sitting less and moving more: the impact of physical activity on mortality in the population of Spain. BMC Public Health 24:3140. doi: 10.1186/s12889-024-20600-y, PMID: 39533197 PMC11559187

[ref9] Deng X. Liu D. Li M. He J. Fu Y. (2024). Physical activity can reduce the risk of blood cadmium and blood lead on stroke: evidence from NHANES. Toxicol. Appl. Pharm. 483:116831. doi: 10.1016/j.taap.2024.116831 38266873

[ref10] Eltit F. Assiri A. Garbuz D. Duncan C. Masri B. Greidanus N.. (2017). Adverse reactions to metal on polyethylene implants: highly destructive lesions related to elevated concentration of cobalt and chromium in synovial fluid. J. Biomed. Mater. Res. A 105, 1876–1886. doi: 10.1002/jbm.a.36057, PMID: 28266173

[ref11] Eltit F. Noble J. Sharma M. Benam N. Haegert A. Bell R. H.. (2021). Cobalt ions induce metabolic stress in synovial fibroblasts and secretion of cytokines/chemokines that may be diagnostic markers for adverse local tissue reactions to hip implants. Acta Biomater. 131, 581–594. doi: 10.1016/j.actbio.2021.06.039, PMID: 34192572

[ref12] Eltit F. Wang Q. Wang R. (2019). Mechanisms of adverse local tissue reactions to hip implants. Front. Bioeng. Biotech. 7:176. doi: 10.3389/fbioe.2019.00176, PMID: 31417898 PMC6683860

[ref13] Garbuz D. S. Tanzer M. Greidanus N. V. Masri B. A. Duncan C. P. (2010). The John Charnley award: metal-on-metal hip resurfacing versus large-diameter head metal-on-metal total hip arthroplasty: a randomized clinical trial. Clin. Orthop Relat. 468, 318–325. doi: 10.1007/s11999-009-1029-x PMC2806981 19697090

[ref14] GBD 2019 Stroke Collaborators (2021). Global, regional, and national burden of stroke and its risk factors, 1990-2019: a systematic analysis for the global burden of disease study 2019. Lancet Neurol. 20, 795–820. doi: 10.1016/S1474-4422(21)00252-0 34487721 PMC8443449

[ref15] He J. Li J. Wu S. Wang J. Tang Q. (2023). Accumulation of blood chromium and cobalt in the participants with metal objects: findings from the 2015 to 2018 National Health and nutrition examination survey (NHANES). BMC Geriatr. 23. doi: 10.1186/s12877-022-03710-3, PMID: 36737686 PMC9898935

[ref16] Hussey D. K. McGrory B. J. (2017). Ten-year cross-sectional study of mechanically assisted crevice corrosion in 1352 consecutive patients with metal-on-polyethylene Total hip arthroplasty. J. Arthroplasty 32, 2546–2551. doi: 10.1016/j.arth.2017.03.020 28392135

[ref17] Jacobs J. J. Cooper H. J. Urban R. M. Wixson R. L. Della V. C. (2014). What do we know about taper corrosion in total hip arthroplasty? J. Arthroplasty 29, 668–669. doi: 10.1016/j.arth.2014.02.014, PMID: 24655613

[ref18] Jiang H. Kavlock K. Li Q. Mistry S. Hermes V. Gibbs A.. (2024). Thirty-five years of reporting of sex and race in clinical studies of U.S. FDA-Authorized Orthopaedic Devices. J. Bone Joint Surg. Am. 106, 2009–2016. doi: 10.2106/JBJS.24.00201, PMID: 39265035

[ref19] Ju M. Li Y. Pei J. Xing J. Wu L. Liu H.. (2024). Association between leisure-time physical activity and all-cause mortality among stroke survivors: findings from National Health and nutrition examination survey. J. Phys. Act Health, 1–10. doi: 10.1123/jpah.2024-0287, PMID: 39547217

[ref20] Kim N. Jacobson M. (2024). Outcomes by race and ethnicity following a Medicare bundled payment program for joint replacement. Jama Netw. Open. 7. doi: 10.1001/jamanetworkopen.2024.33962, PMID: 39287943 PMC11409153

[ref21] Kong B. Chen Y. Cheng S. Ma H. Liu Q. Wang Y.. (2023). Physical activity attenuates the association between blood cadmium exposure and cardiovascular disease: findings from the National Health and nutrition examination survey 2007-2018. Environ. Sci. Pollut. R. 30, 81008–81018. doi: 10.1007/s11356-023-27598-7, PMID: 37310601

[ref22] Kumar S. Selim M. H. Caplan L. R. (2010). Medical complications after stroke. Lancet Neurol. 9, 105–118. doi: 10.1016/S1474-4422(09)70266-2, PMID: 20083041

[ref23] Lassalle M. Colas S. Rudnichi A. Zureik M. Dray-Spira R. (2018). Is there a cardiotoxicity associated with metallic head hip prostheses? A cohort study in the French National Health Insurance Databases. Clin. Orthop. Relat. 476, 1441–1451. doi: 10.1097/01.blo.0000533617.64678.69, PMID: 29698302 PMC6259674

[ref24] Liu H. Wang L. Chen C. Dong Z. Yu S. (2022). Association between dietary niacin intake and migraine among American adults: National Health and nutrition examination survey. Nutrients 14. doi: 10.3390/nu14153052, PMID: 35893904 PMC9330821

[ref25] Lv M. Su C. Huang F. Jia X. Zhang J. Wang H.. (2024). Combined impact of elevated C-reactive protein levels and dyslipidemia on stroke: a CHNS prospective cohort study. Front. Public Health 12:1435004. doi: 10.3389/fpubh.2024.1435004, PMID: 39247228 PMC11377318

[ref26] Madsen T. E. Samaei M. Pikula A. Yu A. Carcel C. Millsaps E.. (2022). Sex differences in physical activity and incident stroke: a systematic review. Clin. Ther. 44, 586–611. doi: 10.1016/j.clinthera.2022.02.006 35418311 PMC9195871

[ref27] Manthe J. Cheng K. Y. Bijukumar D. Barba M. Pourzal R. Neto M.. (2022). Hip implant modular junction: the role of CoCrMo alloy microstructure on fretting-corrosion. J. Mech. Behav. Biomed. 134:105402. doi: 10.1016/j.jmbbm.2022.105402, PMID: 36041275 PMC10507884

[ref28] Maradit K. H. Larson D. R. Crowson C. S. Kremers W. K. Washington R. E. Steiner C. A.. (2015). Prevalence of Total hip and knee replacement in the United States. J. Bone Joint Surg. Am. 97, 1386–1397. doi: 10.2106/JBJS.N.01141, PMID: 26333733 PMC4551172

[ref29] Mastel M. Boisvert A. Moore R. Sutherland F. Powell J. (2022). Metallosis following hip arthroplasty: two case reports. J. Med. Case Rep. 16. doi: 10.1186/s13256-022-03336-4, PMID: 35317840 PMC8941771

[ref30] Mittal M. Siddiqui M. R. Tran K. Reddy S. P. Malik A. B. (2014). Reactive oxygen species in inflammation and tissue injury. Antioxid. Redox Sign. 20, 1126–1167. doi: 10.1089/ars.2012.5149, PMID: 23991888 PMC3929010

[ref31] Moroni A. Savarino L. Hoque M. Cadossi M. Baldini N. (2011). Do ion levels in hip resurfacing differ from metal-on-metal THA at midterm? Clin. Orthop. Relat 469, 180–187. doi: 10.1007/s11999-010-1424-3, PMID: 20544315 PMC3008887

[ref32] Nam D. Li K. Riegler V. Barrack R. L. (2016). Patient-reported metal allergy: a risk factor for poor outcomes after Total joint arthroplasty? J. Arthroplasty 31, 1910–1915. doi: 10.1016/j.arth.2016.02.016, PMID: 26965589

[ref33] Nuevo-Ordonez Y. Montes-Bayon M. Blanco-Gonzalez E. Paz-Aparicio J. Raimundez J. D. Tejerina J. M.. (2011). Titanium release in serum of patients with different bone fixation implants and its interaction with serum biomolecules at physiological levels. Anal. Bioanal. Chem. 401, 2747–2754. doi: 10.1007/s00216-011-5232-8, PMID: 21785984

[ref34] Pang L. Zheng Z. Su P. Xu Z. Chen Y. Liao Z.. (2024). Mendelian randomization of stroke risk after total hip and knee replacements. Front Genet. 15:1435124. doi: 10.3389/fgene.2024.1435124, PMID: 39055256 PMC11270026

[ref35] Rasouli M. R. Tabatabaee R. M. Maltenfort M. G. Chen A. F. (2016). Acute stroke after total joint arthroplasty: a population-based trend analysis. J. Clin. Anesth. 34, 15–20. doi: 10.1016/j.jclinane.2016.03.034 27687339

[ref36] Rees-Punia E. Evans E. M. Schmidt M. D. Gay J. L. Matthews C. E. Gapstur S. M.. (2019). Mortality risk reductions for replacing sedentary time with physical activities. Am. J. Prev. Med. 56, 736–741. doi: 10.1016/j.amepre.2018.12.006, PMID: 30905483 PMC8485344

[ref37] Richardson M. K. Liu K. C. Mayfield C. K. Kistler N. M. Christ A. B. Heckmann N. D. (2023). Complications and safety of simultaneous bilateral Total knee arthroplasty: a patient characteristic and comorbidity-matched analysis. J. Bone Joint Surg. Am. 105, 1072–1079. doi: 10.2106/JBJS.23.00112, PMID: 37418542

[ref38] Roerecke M. (2021). Alcohol's impact on the cardiovascular system. Nutrients 13:419. doi: 10.3390/nu13103419, PMID: 34684419 PMC8540436

[ref39] Rost N. S. Brodtmann A. Pase M. P. van Veluw S. J. Biffi A. Duering M.. (2022). Post-stroke cognitive impairment and dementia. Circ. Res. 130, 1252–1271. doi: 10.1161/CIRCRESAHA.122.319951, PMID: 35420911

[ref40] Sacco R. L. Kasner S. E. Broderick J. P. Caplan L. R. Connors J. J. Culebras A.. (2013). An updated definition of stroke for the 21st century: a statement for healthcare professionals from the American Heart Association/American Stroke Association. Stroke 44, 2064–2089. doi: 10.1161/STR.0b013e318296aeca, PMID: 23652265 PMC11078537

[ref41] Sangani K. Ramesh G. Chakrapani A. (2024). Metallosis following non-metal-on-metal hip arthroplasty: a case report and review. J. Orthop. Case Rep. 14, 111–115. doi: 10.13107/jocr.2024.v14.i09.4748, PMID: 39253647 PMC11381051

[ref42] Santana D. C. Emara A. K. Orr M. N. Klika A. K. Higuera C. A. Krebs V. E.. (2020). An update on venous thromboembolism rates and prophylaxis in hip and knee arthroplasty in 2020. Med. Lithuania 56:416. doi: 10.3390/medicina56090416, PMID: 32824931 PMC7558636

[ref43] Schmid D. Ricci C. Baumeister S. E. Leitzmann M. F. (2016). Replacing sedentary time with physical activity in relation to mortality. Med. Sci. Sport Exer. 48, 1312–1319. doi: 10.1249/MSS.0000000000000913, PMID: 26918559

[ref44] Singh J. A. Yu S. Chen L. Cleveland J. D. (2019). Rates of Total joint replacement in the United States: future projections to 2020-2040 using the National Inpatient Sample. J. Rheumatol. 46, 1134–1140. doi: 10.3899/jrheum.170990, PMID: 30988126

[ref45] Song X. Meng J. Li J. Shen B. Li J. Xu M.. (2024). Association of plasma metals with resting-state functional connectivity in ischemic stroke. Neurotoxicology 104, 56–65. doi: 10.1016/j.neuro.2024.07.011, PMID: 39059632

[ref46] Tian X. Xue B. Wang B. Lei R. Shan X. Niu J.. (2022). Physical activity reduces the role of blood cadmium on depression: a cross-sectional analysis with NHANES data. Environ. Pollut. 304:119211. doi: 10.1016/j.envpol.2022.119211, PMID: 35341822

[ref47] Vrgoc G. Japjec M. Gulan G. Ravlic-Gulan J. Marinovic M. Bandalovic A. (2014). Periprosthetic infections after total hip and knee arthroplasty--a review. Coll. Antropol. 38, 1259–1264. 25842772

[ref48] Wafa H. A. Wolfe C. Emmett E. Roth G. A. Johnson C. O. Wang Y. (2020). Burden of stroke in Europe: thirty-year projections of incidence, prevalence, deaths, and disability-adjusted life years. Stroke 51, 2418–2427. doi: 10.1161/STROKEAHA.120.029606, PMID: 32646325 PMC7382540

[ref49] Wang S. Qin M. Fan X. Jiang C. Hou Q. Ye Z.. (2024). The role of metal ions in stroke: current evidence and future perspectives. Ageing Res. Rev. 101:102498. doi: 10.1016/j.arr.2024.102498, PMID: 39243890

[ref50] Wyles C. C. Wright T. C. Bois M. C. Amin M. S. Fayyaz A. Jenkins S. M.. (2017). Myocardial cobalt levels are elevated in the setting of Total hip arthroplasty. J. Bone Joint Surg. Am. 99:e118. doi: 10.2106/JBJS.17.00159, PMID: 29135673

[ref51] Xiao Y. Yuan Y. Liu Y. Yu Y. Jia N. Zhou L.. (2019). Circulating multiple metals and incident stroke in Chinese adults. Stroke 50, 1661–1668. doi: 10.1161/STROKEAHA.119.025060, PMID: 31167624 PMC6594729

[ref52] You Y. Chen Y. Zhang Y. Zhang Q. Yu Y. Cao Q. (2023). Mitigation role of physical exercise participation in the relationship between blood cadmium and sleep disturbance: a cross-sectional study. BMC Public Health 23:1465. doi: 10.1186/s12889-023-16358-4, PMID: 37525176 PMC10391747

[ref53] Yuan Y. Xiao Y. Feng W. Liu Y. Yu Y. Zhou L.. (2017). Plasma metal concentrations and incident coronary heart disease in Chinese adults: the Dongfeng-Tongji cohort. Environ. Health Persp. 125:107007. doi: 10.1289/EHP1521, PMID: 29064788 PMC5933370

[ref54] Zhong Q. Pan X. Chen Y. Lian Q. Gao J. Xu Y.. (2024). Prosthetic metals: release, metabolism and toxicity. Int. J. Nanomed. 19, 5245–5267. doi: 10.2147/IJN.S459255, PMID: 38855732 PMC11162637

[ref55] Zhu W. Liu Y. Wang W. Zhou Z. Gu J. H. Zhang Z.. (2021). A paradox: Fe2+−containing agents decreased ROS and apoptosis induced by CoNPs in vascular endothelial cells by inhibiting HIF-1alpha. Biosci. Rep. 41:456. doi: 10.1042/BSR20203456, PMID: 33345265 PMC7796189

